# Tuning the Dielectric and Microwaves Absorption Properties of N-Doped Carbon Nanotubes by Boron Insertion

**DOI:** 10.3390/nano11051164

**Published:** 2021-04-29

**Authors:** Qingya Sun, Xinfang Zhang, Ruonan Liu, Shaofeng Shen, Fan Wu, Aming Xie

**Affiliations:** School of Mechanical Engineering, Nanjing University of Science & Technology, Nanjing 210094, China; 13687100556@163.com (X.Z.); liuruonan0420@sina.com (R.L.); frank444111@gmail.com (S.S.); wufan@njust.edu.cn (F.W.)

**Keywords:** microwave absorption, boron doping, reflection loss, effective absorption bandwidth

## Abstract

It is of great significance to regulate the dielectric parameters and microstructure of carbon materials by elemental doping in pursuing microwave absorption (MA) materials of high performance. In this work, the surface electronic structure of N-doped CNTs was tuned by boron doping, in which the MA performance of CNTs was improved under the synergistic action of B and N atoms. The B,N-doped carbon nanotubes (B,N-CNTs) exhibited excellent MA performance, where the value of minimum reflection loss was −40.04 dB, and the efficient absorption bandwidth reached 4.9 GHz (10.5–15.4 GHz). Appropriate conductance loss and multi-polarization loss provide the main contribution to the MA of B,N-CNTs. This study provides a novel method for the design of CNTs related MA materials.

## 1. Introduction

The rapid development of electronic technology has brought great convenience to people, but the electromagnetic interference and electromagnetic pollution brought along with it seriously endanger the life and health of humans [1,2,3,4,5]. In order to solve these problems, it is an urgent task to develop efficient microwave absorption (MA) materials at present. In the past few decades, there have been extensive efforts to construct MA materials with a simple synthesis process which are cost-effective for a long time [6,7,8,9]. In recent years, a series of materials have been designed to convert electromagnetic energy into other forms of energy such as heat. Compared with traditional metallic materials, carbon nanotubes (CNTs), typical carbon nanomaterial, have been widely used in the MA field due to their advantages of unique microstructure, being lightweight, being tunable with the doping of elements, and good electrical conductivity [10,11,12,13,14,15]. Theoretically, the microstructure, electrical conductivity, permittivity, and permeability of CNTs are the key parameters to determine the electromagnetic absorbing property. Therefore, it is an effective way to enhance the MA properties, which uses a method to control these appropriate parameters [16,17,18]. The results show that it is a useful method to regulate the dielectric constant of carbon material when some atoms are doped into the carbon nanostructure [19,20].

Up to now, the introduction of N, S, and other elements into carbon materials has been widely used. Especially in electromagnetic absorption, the N atom doping can regulate the conductivity of CNTs, thus promoting impedance matching and finally enhancing MA properties [21,22,23,24,25,26,27]. The Co-C/MWCNTs composites were successful prepared with N-doped. It is found that C atoms have different electronegativity from doped N heteroatoms, which lead to the formation of dipole polarization and thus enhanced the dielectric loss capacity of a material [28]. Compared with single element doping, the carbon materials with multi-components doped show better impedance matching and higher dielectric loss [29]. By doping Co/N outer shell in the carbon, it is found that the minimum value of reflection loss reached the −52.5 dB at 13.1 GHz with the thickness of 2.2 mm, and the effective absorption bandwidth over −10 dB was 4.4 GHz [22]. The literature suggests that cooping of B can form a nanojunction in graphene. In this process, the lattice structure of graphene changes when a B atom replaces carbon in the lattice of graphene and forms a stable covalent bond with adjacent C atoms, which results in the tuning of electronic structure [30,31,32]. The doping of B can effectively improve the electronic properties and energy storage performance of graphene, which has been widely studied in electrocatalytic activity [33]. At present, the CNTs doped by B atom have not been thoroughly studied as MA materials. It is the obvious difference in electronegativity between C (2.5), B (2.0) that is reasonably believed to result in uneven charge distribution, which can generate defects in CNT [31]. Due to the existence of mutual electronic structure and similar atomic size between C, B, and N atoms, each two of which can form stable covalent bonds [30]. Therefore, the synergistic effect of the co-doping of B and N atoms on the MA performance of CNTs needs to be further investigated.

Inspired by these advantages, the dielectric parameters of MA materials are tuned by doping B atoms to regulate the surface electronic structure of N-doped CNTs. Recently, it has been found that melamine can effectively synthesize CNTs [34]. In this work, the N-doped CNTs were one-step synthesized in situ by high-temperature pyrolysis using melamine as a carbon source. In this process, a proper amount of boric acid was added in order to achieve effective doping of B atoms. When the B,N-CNTs were evaluated as absorbers, they showed high MA performance, in which the lowest reflection loss value of MA material reached −40.04 dB and the effective absorption bandwidth exceeding −10 dB and ranged from 10.5 to 15.4 GHz. The doping of B atoms could increase the defects of CNTs, and the doping of N atoms could control the dielectric of carbon materials, which promotes the formation of polarization loss and conductance loss. It is believed to be the main mechanism for improving MA performance. This work suggests that the novel B,N-CNTs with lightweight and good MA performance could be an effective candidate material for absorbers applications.

## 2. Experimental Section

### 2.1. Materials

Nickel chloride hexahydrate (NiCl_2_∙6H_2_O, 99%), melamine (99%), boric acid (H_3_BO_3_, 99.5%), and ethanol (95%) were provided by Titan Scientific Co., Ltd. (Shanghai, China).

### 2.2. Synthesis of B,N-CNTs

The B,N-CNTs precursors were synthesized as follows. One gram of melamine and 0.7 g of NiCl_2_∙6H_2_O and boric acid of different qualities were mixed in ethanol (50 mL), in which the addition amount of boric acid was 0.05, 0.1, 0.25, 0.5, and 1 g, respectively. Then, the mixed solutions were placed on the rotary evaporator for solute separation, and the B,N-CNTs precursors were obtained. After that, the precursor was collected into a tube furnace; the internal ambient temperature of the tube furnace chamber was gradually heated to 700 °C by 10 °C/min under an Ar atmosphere and then held for 3 h. The mixture of melamine and boric acid was transformed into B,N-CNTs via a nickel (Ni)-assisted pyrolysis [34]. Finally, sulfuric acid (1 M) was used to saturate the carbide for removing Ni particles in the carbon nanostructure, which the saturating time was 6 h. As the addition amount of boric acid was 0.05, 0.1, 0.25, 0.5, and 1 g, the B, N-doped carbon nanotubes were named B,N-CNT-1, B,N-CNT-2, B,N-CNT-3, B,N-CNT-4, and B,N-CNT-5, respectively.

### 2.3. Characterization and Measurement

The morphology of B,N-CNTs were observed on a transmission electron microscope (TEM, FEL F20, 200 kV, Lincoln, NE, USA), a high-resolution transmission electron microscopy (HRTEM, Tecnai G^2^ F20, FEI, Lincoln, NE, USA), and a scanning electron microscopy (SEM, Nova Nano 450, FEI, Lincoln, NE, USA) with an energy dispersive spectrometer (EDS, FEI, Lincoln, NE, USA). The crystal structure was characterized by X-ray diffraction (XRD, X′Pert-PRO, PANalytical, Almelo, NL, USA), which used a Cu Kα radiation source (40 kV, 30 mA). X-ray photoelectron spectroscopy (XPS) was measured on an apparatus (ESCALAB 250Xi, Waltham, MA, USA). The vector network analyzer (VAN, N5244A PNA-X, Santa Rosa, CA, USA) was used to measure complex permittivity (*ε*_r_) and complex permeability (*μ*_r_) in the frequency range from 2 to 18 GHz. The materials with a mass percentage of 10 wt% were added into wax for evenly mixing and then pressed into cylindrical samples with an inner diameter of 3.04 mm and an outer diameter of 7 mm.

## 3. Results and Discussion

**Figure 1:**
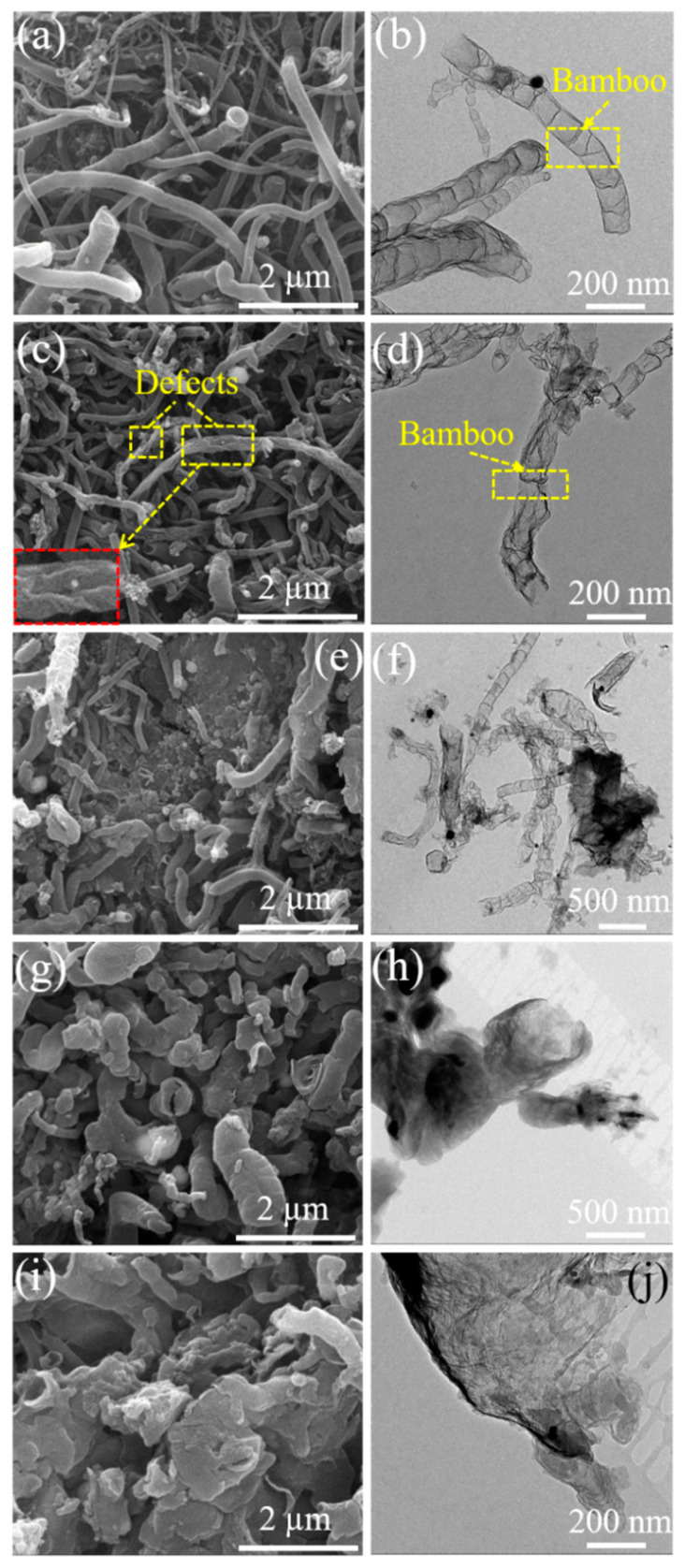
SEM and TEM images of B,N-CNT-1 (**a**,**b**), B,N-CNT-2 (**c**,**d**), B,N-CNT-3 (**e**,**f**), B,N-CNT-4 (**g**,**h**), and B,N-CNT-5 (**i**,**j**).

Figure 1 shows the SEM and TEM images of B,N-CNTs at different addition amounts of boric acid. As the addition amount of boric acid is 0.05 g, it can be observed that the surface of CNTs is smooth and grows into the bamboo shape (Figure 1a,b). The diameter and length of B,N-CNT-1 were measured to be about 100 nm and dozens of micrometers. In Figure 1c,d, the most structures of B,N-CNT-2 were transformed and had many defects with the addition amount of boric acid increase to 0.1 g; many pits appeared in the wall of B,N-CNTs, which caused the tubular shape to become slightly twisted and wrinkled. However, the size of B,N-CNTs did not appear to change significantly. For the morphology of B,N-CNT-3, it could be seen that a part of CNTs had not been fully grown, and the size was nonuniform, which the CNTs lengths decreased to several micrometers (Figure 1e,f). It indicates that the addition of B element hindered the growth of CNTs. As the addition amount of boric acid increase to 0.5 g, the bamboo shape disappeared in the B,N-CNT-4, the size of the nanotube wall became thick, growth length of CNT became relatively short, only a few hundred nanometers, and the diameter increased significantly to about 500 nm (Figure 1g,h). In Figure 1i,j, the many carbon nanostructures can be seen transformed from a tubular shape to a sheet shape with the amount of boric acid increased to 1 g. The mapping morphology of B,N-CNT-5 is shown in Figure S1a–f (Supplementary Materials); the element B and N were clearly characterized in the carbon nanostructure. According to the atomic percentage data, it was found that B accounts for 12.58%, N for 6.3%, and C for 74.95%. It indicates that elements B and N were successfully doped into the CNTs. Therefore, a small amount of doping of B could lead to defects on the surface of CNTs, which will affect the growth of CNTs when the content of B is high.

**Figure 2:**
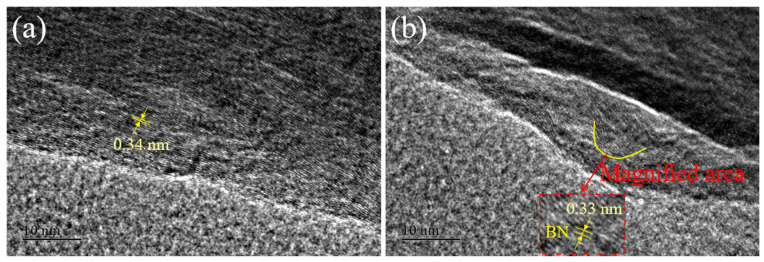
HRTEM images of carbon (**a**) and BN (**b**) in the B,N-CNT-4.

To further demonstrate the morphology of the samples, HRTEM images of B,N-CNT-4 are shown in Figure 2. The interlayer distance of the material was measured to be 0.34 nm in (002) plane, which corresponded to a typical lattice spacing of graphitic carbon (Figure 2a). It indicated that the degree of graphitization of B,N-CNT was reasonable. In addition, the nanodomain can be seen in Figure 2b, where the lattice spacing was 0.33 nm with multiple amplification of the image, which was expressed as B [35].

In Figure S2, the crystal structures of B,N-CNTs were characterized by XRD patterns. Only one characteristic peak was seen in the five cures, which represented the (002) crystal plane of hexagonal carbon and BN at 26.1° [35], which indicated that B and N atoms had been doped into the carbon nanostructure and there were no other impurities in the sample. Through the comparison of all curves of the XRD pattern, it was observed that the intensity of the diffraction peak increased in turn with the increase in B content due to the increase in B atoms during the CNTs growth process, which could adsorb more N atoms in melamine, leading to the increase in BN content.

**Figure 3:**
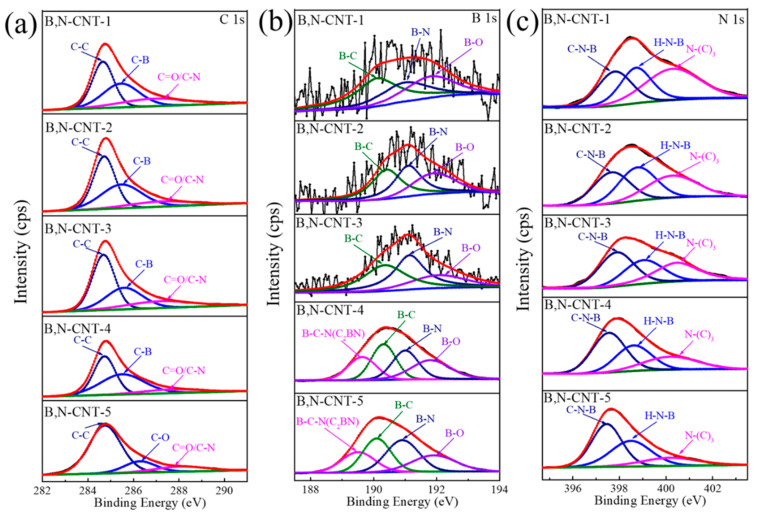
The (**a**) C 1s spectrum, (**b**) B 1s spectrum, and (**c**) N 1s spectrum of B,N-CNTs.

As shown in Figure 3, the surface chemical compositions of B,N-CNTs were measured by XPS. Figure S3 (Supplementary Materials) exhibits the survey spectrum of B,N-CNTs, the peaks of B, C, and N elements are clearly represented on the spectrum curve of materials. As B increased, the strength of B and N elements increased, while the strength of C element decreased due to the addition of B reducing the crystallinity of carbon material. By contrast, morphology characterization and XRD analysis indicated that B atoms and N atoms had been successfully doped and simultaneously incorporated into the carbon nanostructure. In order to analyze the bonding between C, B, and N, the C 1s spectrum, B 1s spectrum, and N 1s spectrum of B,N-CNTs were characterized in detail. In Figure 3a, three different peaks with binging energy of 284.7, 285.5, and 288.1 eV are shown in C 1s spectrum of B,N-CNT-1, 2, 3, and 4, which represent at C-C, C-B, and C=O/C-N groups, respectively [36]. The peak at 286.2 of B,N-CNT-5 was attributed to C-O bonds [36]. It was found that the area of C=O/C-N groups decreased gradually with the increase in B, while for the rest of the areas, bonding configurations changed little. For B 1s spectrum of B,N-CNT-1, B,N-CNT-2, and B,N-CNT-3, three bonding configurations were displayed at 190.1, 190.9, and 191.9 eV, which were attributed to B-C [33], B-N, and B-O groups, respectively (Figure 3b) [36]. As the content of B increased from 0.5 to 1 g, the B-C-N (C_2_BN) group with the peak of 189.6 appeared in the B 1s spectrum. By calculation, the area of B-C groups of B,N-CNT-1, B,N-CNT-2, B,N-CNT-3, B,N-CNT-4, and B,N-CNT-5 was obtained at 156, 159, 334, 947, and 1950 respectively, and the B-N group area of every B,N-CNT was 103, 164, 367, 874 and 2138 respectively, showing a rising trend. A comparison between B,N-CNT-1 and B,N-CNT-2 showed that the area ratio of B-N group decreased and the area ratio of the B-C group increased in the B 1s spectrum when the content of B decreased from 0.1 to 0.05 g. It indicated that the amount of B was low, the C atoms in the material are first taken away by B atoms, and thus the B-C groups were formed. However, the B-N groups began to become more numerous and dominant with the continuous increase in B content. The N 1s spectrum of B,N-CNTs was observed in Figure 3c; it was found that three binding energy peaks located at 397.4, 398.5, and 400.1 eV, which represented C-N-B, H-N-B and N-(C)_3_ groups, respectively [37]. The area of the C-N-B group increased and the area of N-(C)_3_ group decreased with the content of B increased, consistent with the trend of the curve in the C 1s spectrum and B 1s spectrum. The atomic ratios of B, N, and C in B,N-CNTs samples are presented in Figure S4 (Supplementary Materials); with decreased B content, the B atomic percent showed an upward trend, the N atomic percent first decreased and then increased, the C atomic percent showed a trend of increasing and then decreasing; consistent with the variation of B-C and B-N groups in the B,N-CNTs.

The reflection loss (*RL*) of carbon nanomaterial can be used to characterize its MA property in the transmission-line theory, and the calculation formulas are shown in Equations (1) and (2) [38]:

(1) $$RL\left(\mathrm{dB}\right)=20\log\left|\frac{Z-1}{Z+1}\right|$$

(2) $$Z=\frac{Z_{in}}{Z_{0}}=\sqrt{\frac{\mu_{r}}{\varepsilon_{r}}}\tan\;h\left(j\frac{2\pi fd}{c}\sqrt{\varepsilon_{r}\mu_{r}}\right)$$

where *Z_in_* represents the input impedance of MA materials, *Z*_0_ stands for the impedance of free space, *ε_r_* (*ε_r_ = ε′ − jε″*) represents complex permittivity, *μ_r_* (*μ_r_ = μ′ − jμ″*) stands for complex permeability, *f* stands for frequency, *d* is the thickness of MA materials, and c stands for the light speed.

**Figure 4:**
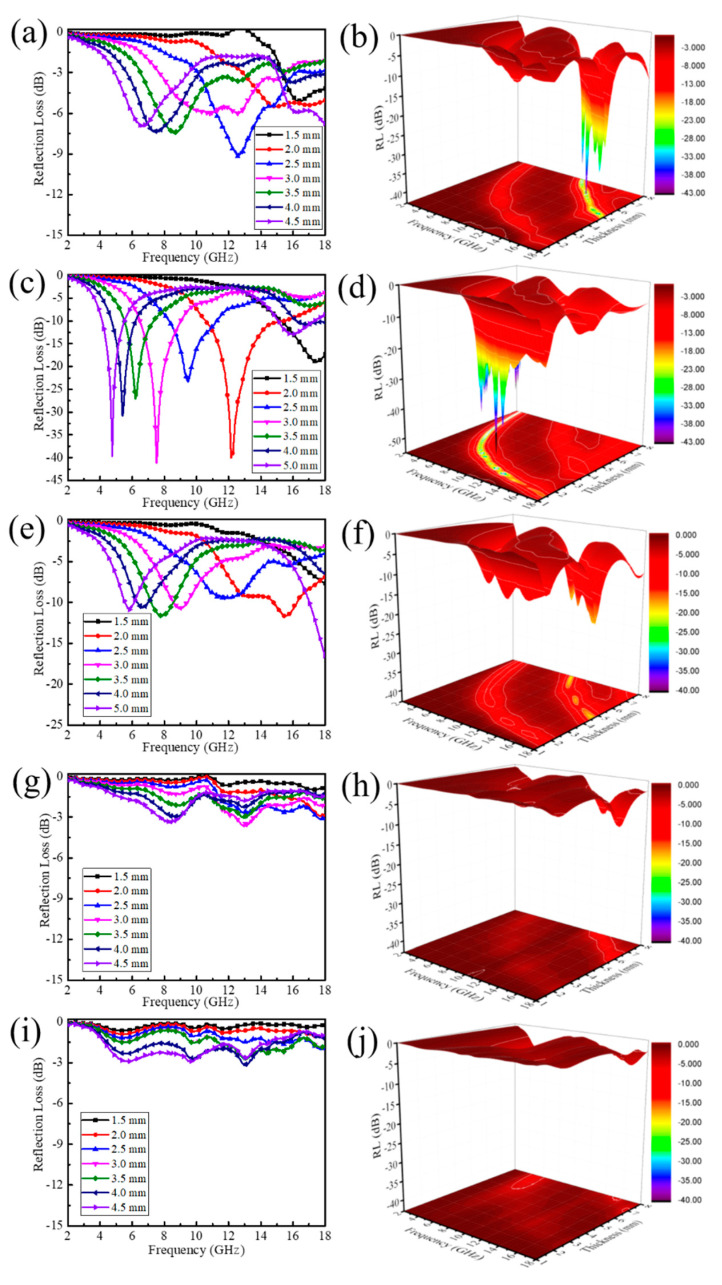
Reflection Loss curves and 3D plot of B,N-CNT-1 (**a**,**b**), B,N-CNT-2 (**c**,**d**), B,N-CNT-3 (**e**,**f**), B,N-CNT-4 (**g**,**h**), and B,N-CNT-5 (**i**,**j**) with a mass percentage of 10 wt% in wax.

Research has shown that more than 90% of incident electromagnetic waves can be attenuated as the RL value of MA materials is below −10 dB, and the frequency range below −10 dB is considered as the effective absorption bandwidth. As shown in Figure 4, the RL curves with a three-dimensional *RL* of the prepared B,N-CNTs in the frequency range of 2–18 GHz was analyzed. The MA property of B,N-CNT-1, presented in Figure 4a,b, shows that the minimum RL value was −9.2 dB in the frequency region from 2 to 18 GHz, which suggests that B,N-CNT-1 could not be practically applied in the field of MA materials. When B content increased to 0.1 g, the MA performance of B,N-CNT was significantly enhanced, in which the B,N-CNT-2 with thickness of 2 mm had the best MA performance (Figure 4c,d). It was exhibited that the minimum *RL* reached −40.04 dB at 12.2 GHz and the effective absorption bandwidth was from 10.5 to 15.4 GHz. The improvement of MA performance of B,N-CNT-2 was mainly caused that the appropriate addition of B element led to more defects on the surface of B,N-CNT and thus increased the interfacial polarization of the absorbent. Compared with the *RL* curve of B,N-CNT-2, it was clearly shown that the lowest RL of B,N-CNT-3 was increased to −16.5 dB at 18 GHz, and the effective absorption bandwidth was only 1 GHz with a thickness of 5 mm in Figure 4e,f. As shown in Figure 4g–j, with the B content increased from 0.5 to 1 g, the RL values under all thickness of the B,N-CNTs were lower than −4 dB, which indicates that they were not suitable for application as absorbents.

**Figure 5:**
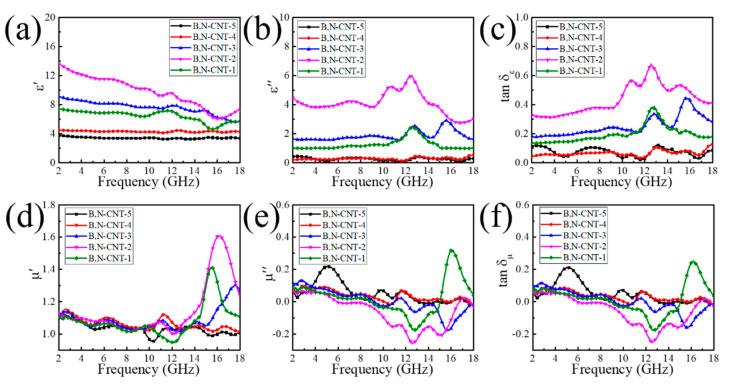
*ε′* (**a**), *ε″* (**b**), tan *δ_ε_* (**c**), *μ′* (**d**), *μ″* (**e**), and tan *δ*_μ_ (**f**) of B,N-CNTs.

The complex permeability and permittivity are the main reasons that affect the MA property of B,N-CNTs, which the magnetic loss ($\tan\;\delta_{\mu }=\mu^{\prime \prime }/\mu^{\prime }$) and dielectric loss ($\tan\;\delta_{\varepsilon }=\varepsilon^{\prime \prime }/\varepsilon^{\prime }$) were measured at the frequency range from 2 to 18 GHz. As is known, the storage ability of magnetism and electromagnetic are represented by the real parts of permeability (*μ′*) and permittivity (*ε′*), and the dissipation ability of magnetism and electromagnetic were related to imaginary parts of permeability (*μ″*) and permittivity (*ε″*). As shown in Figure 5a–c, it was found that the *ε′*, *ε″* and *tanδ*_ε_ of B,N-CNT-2 were higher than that of other MA materials, and the storage ability of electromagnetic of B,N-CNT-2 gradually decreased with an increase in frequency from 2 to 16 GHz, but the opposite trend was in the frequency range of 16–18 GHz. In addition, it was found that two distinct peaks (5.3 at 10.68 GHz and 6 at 12.4 GHz) were in the *ε″* curve of B,N-CNT-2, which corresponded to the second half of the fluctuation region in *ε′* curve. Therefore, it indicates that the polarization resonance reactions occurred in two frequency ranges as determined from the Debye theory equation [17]. In Figure 5d–f, it can be observed that the *μ′* values of B,N-CNTs were below 1.12 in the frequency range of 2–13.8 GHz, which the storage ability of magnetism of B,N-CNT-1, 2, and 3 significantly enhanced in the high frequency range (13.8–18 GHz), the rest of curves did not change much. The minimum *μ″* values of B,N-CNT-1, 2, and 3 were −0.18 at 12.8 GHz, −0.26 at 12.6 GHz, and −0.18 at 15.64 GHz, respectively. The values of *μ″* were below zero, indicating that the magnetic energy was radiated out from B,N-CNTs/paraffin and transformed into electric energy [39]. As the effect of excessive doping of B changing the MA performance of B,N-CNTs was low, the *μ″* values of B,N-CNT-4 and 5 could be ignored. By comparing tan *δ_ε_* and tan *δ_μ_*, it was found that magnetic loss was less than dielectric loss. Therefore, the dielectric loss was dominant in the MA mechanism.

The impedance matching (|*Z*|) and attenuation constant (*α*) are also the key factors to determine the MA performance of materials, which the calculation equations are presented in (3) and (4).

(3) $$\left|Z\right|=\left|\sqrt{\frac{\mu_{r}}{\varepsilon_{r}}}\right|$$

(4) $$\alpha =\frac{\sqrt{2}\pi f}{c}\times \sqrt{\left(\mu^{\prime \prime }\varepsilon^{\prime \prime }-\mu^{\prime }\varepsilon^{\prime }\right)+\sqrt{{\left(\mu^{\prime \prime }\varepsilon^{\prime \prime }-\mu^{\prime }\varepsilon^{\prime }\right)}^{2}+{\left(\mu^{\prime }\varepsilon^{\prime }+\mu^{\prime \prime }\varepsilon^{\prime \prime }\right)}^{2}}}$$

**Figure 6:**
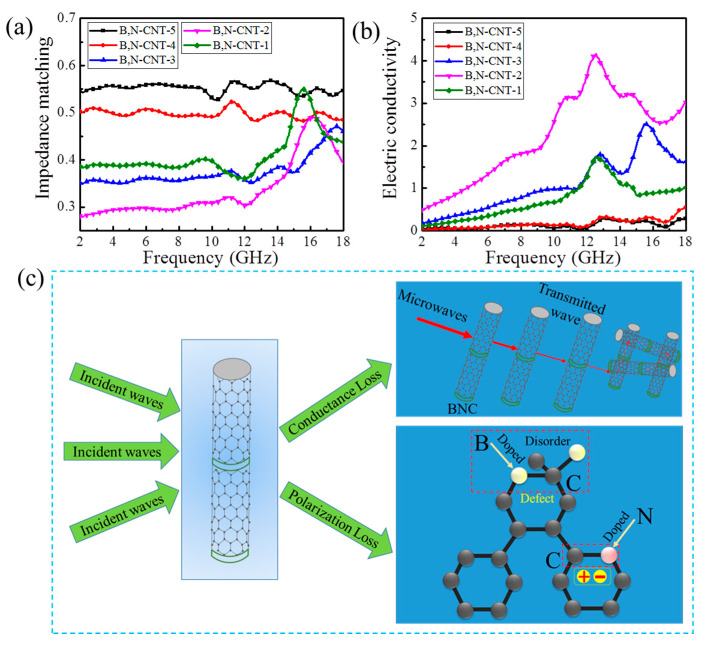
The |*Z*| curves (**a**), Electric Conductivity curves (**b**), and illustration of the MA performance of B,N-CNTs (**c**).

It is well known that the closer the |*Z*| value is to 1, the greater the matching degree between the material and electromagnetic microwave will be, and thus more incident microwaves will enter the absorbent. The *|Z|*, *α* and electrical conductivity of B,N-CNTs are shown in Figure 6, the |*Z*| values of B,N-CNT-1 were higher than B,N-CNT-2 (Figure 6a), it was due to the content of B being 0.05 g, most of the B atoms combined with C atoms to form the B-C group in B,N-CNTs, and B was the permeable microwave element. However, when the content of B increased to 0.1 g, the B atoms began to combine with N atoms of B,N-CNT-2 in large quantities, and the disappearance of N atoms in CNTs reduced the α and electrical conductivity. Therefore, the α and electrical conductivity values of B,N-CNT-1 were lower than B,N-CNT-2 (Figure S5 and Figure 6b). With the increase in B content again, the area of B-C and B-N groups became larger, leading to the increase in |Z| values of B,N-CNTs, and α and electrical conductivity decreased. After mutual balance between impedance matching, dielectric loss, and magnetic loss, we found that the B,N-CNT-2 had higher MA performance, while the conductance loss and polarization relaxation were the main MA mechanisms. The relaxation phenomenon and conductance loss were considered to be the main factors affecting the MA performance of absorbents. The MA mechanism of B,N-CNTs was further analyzed by Debye theory, which is expressed as Equations (5) and (6) [40]:

(5) $$\varepsilon^{\prime }=\varepsilon_{\infty }+\frac{\varepsilon_{s}-\varepsilon_{\infty }}{1+\omega^{2}\tau^{2}}$$

(6) $$\varepsilon^{\prime \prime }=\frac{\varepsilon_{s}-\varepsilon_{\infty }}{1+\omega^{2}\tau^{2}}\omega \tau +\frac{\sigma }{\omega \varepsilon_{0}}$$

where *ε*_s_ stands for static dielectric constant, *ε*_∞_ stands for the complex dielectric permittivity at the high-frequency limit, *ε*_o_ represents the dielectric constant in a vacuum, *ω* = 2π*f* represents the angular frequency, *τ* represents the relaxation time, and *σ* represents the conductivity. The curve is composed of *ε′* and *ε″* and is called the Cole–Cole curve. The Cole–Cole curve of B,N-CNT-2 can be observed in Figure S6 (Supplementary Materials); it was found that more than three semicircles in the curve and a short straight line appeared at the tail, which indicated that multi-relaxations and conductance loss occurred simultaneously.

As shown in Figure 6c, the MA mechanism of the B,N-CNTs is summarized on the basis of the analysis, which mainly consisted of conductance loss and polarization loss. When the incident microwave enters into CNT, part of the microwave was lost, and the other part could pass through the current CNT to the next one. This nanostructure could extend the propagation path of microwave, and the microwave energy would gradually attenuate under the action of conductance loss. The impedance matching of carbon materials was improved by appropriate doping of B atoms due to B being a microwave-permeable material. On the other hand, B atoms could cause the disordered arrangement of C atoms to form defects in the CNT. It increased the polarization loss effect of the B,N-CNTs. In addition, the doped N atoms could generate dipole polarization on the B,N-CNTs. In a word, the microwave attenuation of B,N-CNTs was mainly caused by conductance loss and multi-polarization loss.

## 4. Conclusions

In summary, B, N-doped carbon nanotubes were prepared, using a one-step in situ synthesis method and searched the MA performance. When the amount of boric acid added was 0.1 g, melamine was carbonized into a corrugated bamboo shape at 700 °C with catalytic Ni. The MA performance and electrical conductivity display an upward trend followed by a downward trend with an increase in B, while the impedance matching was just the opposite. It indicated that the conductance loss and multi-polarization were the dominant MA mechanism. It could be observed that the B,N-CNT-2 achieved the optimal MA property, where the lowest RL reached −40.04 dB with an effective absorption bandwidth of 4.9 GHz. The appropriate doping of B and N atoms not only enhanced the multi-polarization effect of B,N-CNTs but also increased the impedance matching. Therefore, it is believed that B,N-CNTs are of great potential as MA materials.

## Data Availability

The data that support the findings of this study are available from the corresponding author upon reasonable request.

## Supplementary Materials

The following are available online at https://www.mdpi.com/article/10.3390/nano11051164/s1, Figure S1: Element mapping analysis of B,N-CNT-5: SEM image (a), overall element distribution (b), B element (c), N element (d), C element (e) and element content (f). Figure S2: The XRD patterns of B,N-CNTs. Figure S3: The survey spectrums of B,N-CNTs. Figure S4: The atomic ratios of B (a), N (b) and C (c) in B,N-CNTs. Figure S5: The Attenuation Constant curves of B,N-CNTs. Figure S6: The Cole-Cole curve of B,N-CNT-2.
