# Supplementary material for: Tuning the Dielectric and Microwaves Absorption Properties of N-Doped Carbon Nanotubes by Boron Insertion
Source: Nanomaterials (Basel). 2021 Apr 29;11(5):1164. doi: 10.3390/nano11051164 (PMC8145881; doi:10.3390/nano11051164)
Supplement: Supplementary file 1 [file nanomaterials-11-01164-s001.zip › nanomaterials-1192640-supplementary.pdf]

Supplementary material

Tuning the Dielectric and Microwaves Absorption Properties of N-Doped Carbon Nanotubes by Boron Insertion

Qingya Sun*, Xinfang Zhang, Ruonan Liu, Shaofeng Shen, Fan Wu, Aming Xie*

School of Mechanical Engineering, Nanjing University of Science and Technology, Nanjing 210094, China

School of Mechanical Engineering, Nanjing University of Science and Technology, Nanjing 210094, China; 13687100556@163.com (X.Z.); liuruonan0420@sina.com (R.L.); frank444111@gmail.com (S.S.); wufan@njust.edu.cn (F.W.)

* Correspondence: sunqingya@njust.edu.cn (Q.S.); xieaming@njust.edu.cn (A.X.)

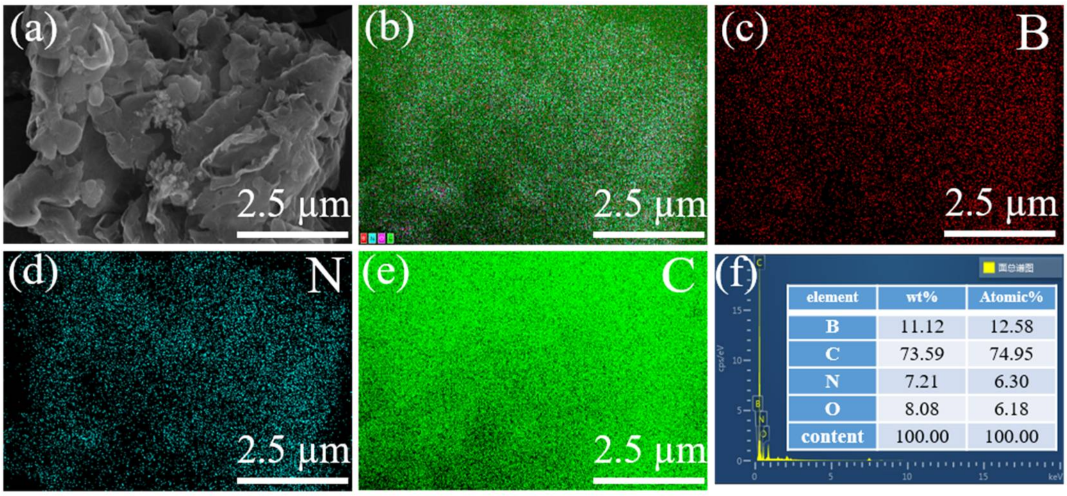

Figure S1. Element mapping analysis of B,N-CNT-5: SEM image (**a**), overall element distribution (**b**), B element (**c**), N element (**d**), C element (**e**) and element content (**f**).

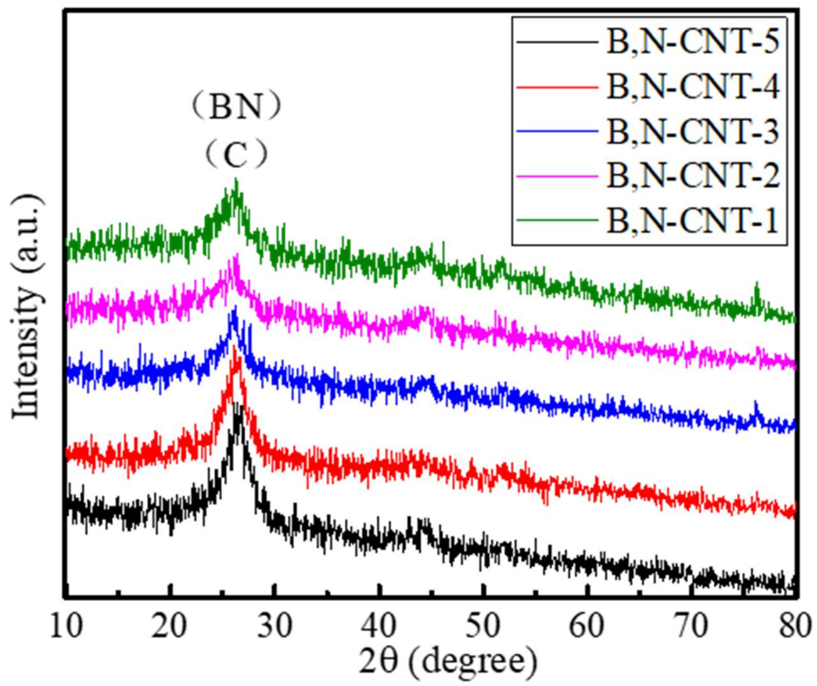

Figure S2. The XRD patterns of B,N-CNTs.

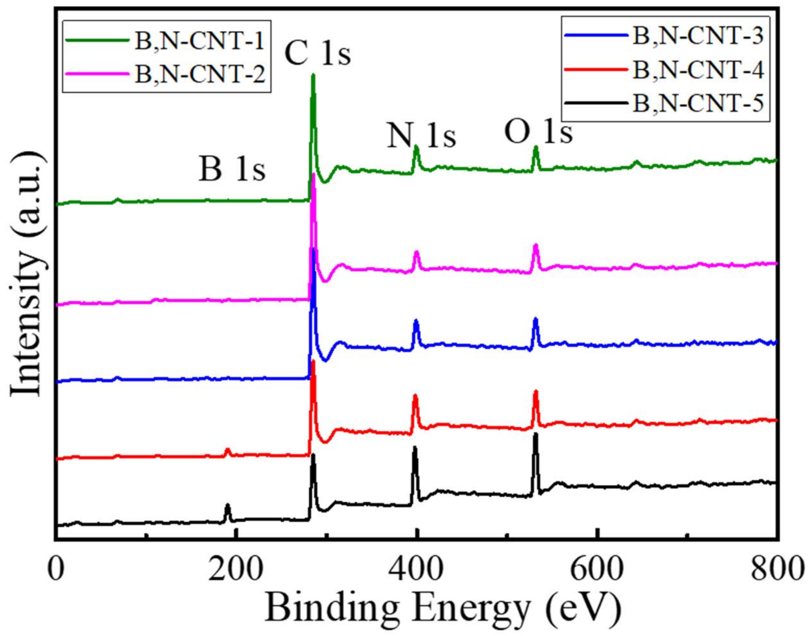

Figure S3. The survey spectrums of B,N-CNTs.

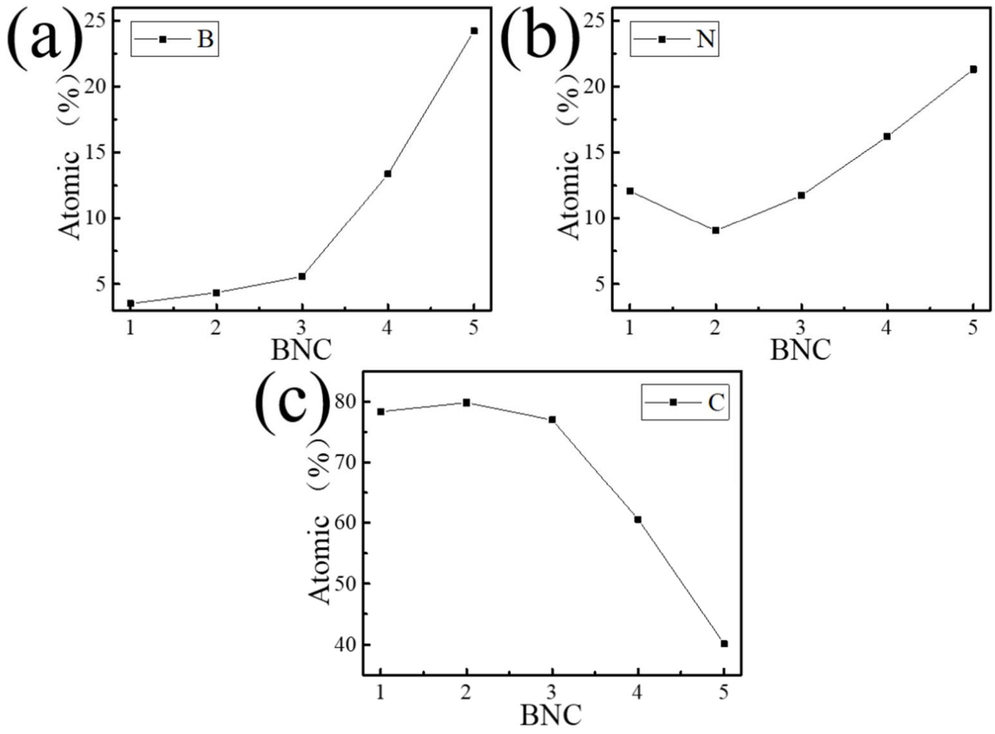

Figure S4. The atomic ratios of B (a), N (b) and C (c) in B,N-CNTs.

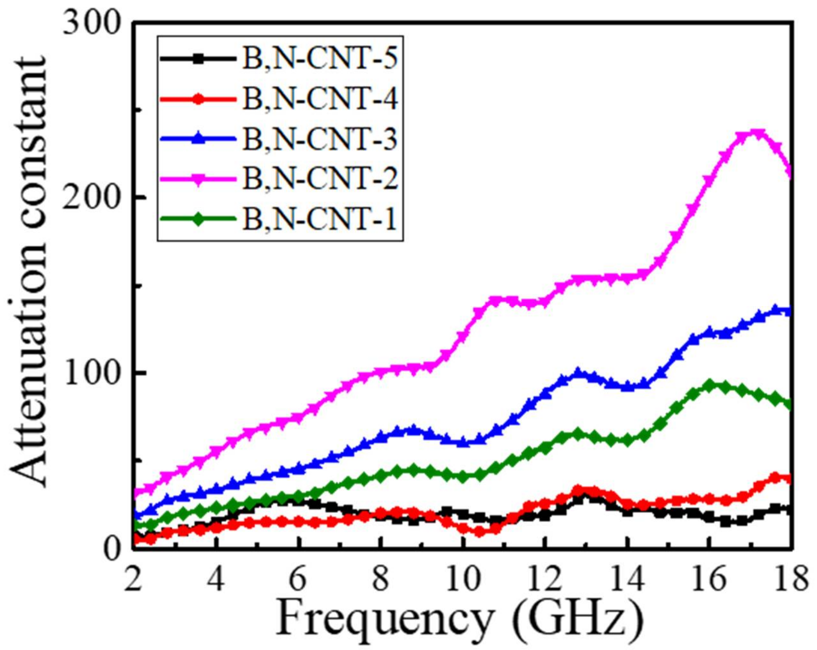

Figure S5. The Attenuation Constant curves of B,N-CNTs.

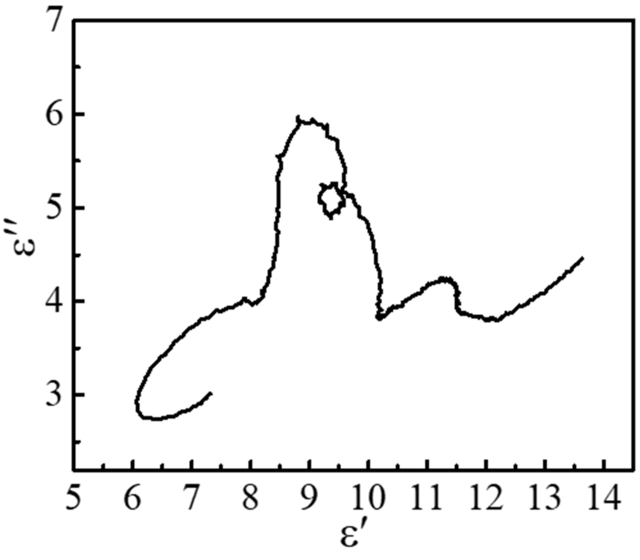

Figure S6. The Cole-Cole curve of B,N-CNT-2.
